# Implementation of Implementation Science Knowledge: The Research‐Practice Gap Paradox

**DOI:** 10.1111/wvn.12403

**Published:** 2019-10-11

**Authors:** Anna Westerlund, Per Nilsen, Linda Sundberg

**Affiliations:** ^1^ Department of Epidemiology and Global Health Umeå University Umeå Sweden; ^2^ Department of Medical and Health Sciences Division of Community Medicine Linköping University Linköping Sweden

A person who wants to find a solution to a public health problem has a different task than someone who wants to create or test a theory. (Eldredge, Markham, Ruiter, Kok, & Parcel, 2016, p. 8)

The challenges in improving health care are considerable, as are the efforts made to develop and deliver best practice (Grol, Wensing, Eccles, & Davis, 2013). Different interventions with evidence of effectiveness are continuously made available for potential improvement of health care. However, the difficulties in implementing and using such evidence are well known (Greenhalgh, Robert, Macfarlane, Bate, & Kyriakidou, 2004). The knowledge‐practice gap in health care refers to the gap between scientific knowledge and its application in routine healthcare practice.

Implementation science has developed in the 2000s in response to this gap, with the ambition to generate knowledge to promote a better uptake of evidence for improvements in the quality and safety of health care. The body of implementation knowledge comprises a rapidly growing amount of empirical studies as well as countless theories, frameworks, and models, contributing to an understanding of factors associated with successful implementation of evidence‐based interventions within a variety of settings (Tabak, Khoong, Chambers, & Brownson, 2012).

The multitude of empirical implementation studies, as well as theories, models, and frameworks developed in implementation science, reflect a growing evidence‐based concerning implementation (Brownson, Colditz, & Proctor, 2018). However, despite the rapid progress of implementation science, the knowledge‐practice gap in health care is still substantial, as shown in studies that describe difficulties in achieving desirable change in healthcare practice. Low rates of adoption and limited use of evidence‐based interventions are persistent problems. Thus, the challenges of reducing the knowledge‐practice gap still remain after more than two decades of research.

The aim of this editorial is to address the knowledge‐practice gap by means of increasing awareness of a parallel knowledge‐practice gap (i.e., the somewhat paradoxical gap between scientific knowledge concerning implementation and actual real‐life implementation and use of this knowledge in healthcare practice).

This editorial is based on findings and conclusions presented in a doctoral thesis by the first author, which investigated the resemblance between available scientific knowledge on implementation and implementation strategies used in healthcare practice in three large improvement efforts in Sweden (Westerlund, 2018). An overall conclusion of the thesis was that there exists a parallel knowledge‐practice gap between scientific knowledge on implementation and the use of this knowledge in implementation efforts in healthcare practice (Westerlund, 2018; Westerlund et al., 2017). The findings showed that implementation knowledge was not transferred to healthcare practice (and practitioners) to a sufficient extent, thus restricting the systematic use of implementation knowledge in practice.

Implementation science has a twofold aim: to produce knowledge sufficiently generalizable to contribute to scientific knowledge accumulation and to produce knowledge applicable for improved practice (Fixsen, Blase, & Van Dyke, 2019). The question of use, applicability, and impact of implementation science has been highlighted previously, and the need to make implementation science knowledge more relevant and widely disseminated has been called for in the literature (Armson, Roder, Elmslie, Khan, & Straus, 2018; McIsaac et al., 2018). Implementation knowledge is not taught in healthcare practitioners’ basic training and only seldom in continuing professional education. Although the literature on evidence‐based implementation is expanding and courses are increasingly being made available, these do not focus on practical issues or guidance on how to actually use implementation science knowledge in implementation endeavors (Nilsen, Neher, Ellström, & Gardner, 2017). Ovretveit, Mittman, Rubenstein, and Ganz (2017) have noted that healthcare practitioners are not expected to be knowledgeable about implementation science.

Although implementation science is widely considered an applied science, the extent to which knowledge produced in this field is actually used by practitioners is not known. There are few empirical studies concerning if or how scientific knowledge on implementation is being used in healthcare practice (Armson et al., 2018). As implementation researchers, we need to ask ourselves if our research findings and evidence on implementation have reached the world of practice to a sufficient degree.

There are many analytical tools aimed at supporting researchers’ use of implementation science in their research endeavors (Simpson et al., 2013). When approaching the implementation knowledge field, phrases such as the following are frequently encountered:“Theories and frameworks enhance implementation research” and”inform study design and execution” (Tabak et al., 2012, p. 6) or“Scholars seeking to study implementation have over 60 conceptual frameworks to guide their work” (Birken et al., 2017, p. 2). The impression is that models and frameworks are developed to“help advance implementation science” (Damschroder et al., 2009, p. 2). Recently, the ImpRes tool was developed with the stated purpose to“support research teams in the process of designing implementation research” (King's Improvement Science, 2018, p. 1). These observations raise the questions of whether other researchers are the primary target audience of implementation science knowledge and the extent to which the knowledge produced in the field actually reaches beyond academia. To a large extent, knowledge produced in implementation science still seems to belong to the scientific community rather than practitioners to improve outcomes in health care (Armson et al., 2018; Ovretveit et al., 2017; Westerlund, 2018).

Considering the vast amount and variation of empirical studies of implementation efforts in many different healthcare settings, there is no question that the field of implementation science has produced knowledge on implementation of great relevance for potential use in health care. It seems highly plausible that a conscious and systematic use of scientific knowledge on implementation would be beneficial in change efforts in health care and would likely increase adoption and use of research‐informed interventions to improve the quality of care. Hence, applying scientific knowledge on implementation in healthcare practice may help bridge the knowledge‐practice gap in health care.

So‐called”action models” such as Knowledge‐to‐Action (Graham et al., 2006) and Quality Implementation Framework (QIF; Meyers, Durlak, & Wandersman, 2012) have been developed to guide the translation of research into practice. The originators of the QIF introduced the concept of”practical implementation science,” which refers not only to the translation of implementation science knowledge into user‐friendly resources but also to research and actions based on this translation. Meyers and colleagues stated that one of their goals was to”outline practical implications for improving future implementation efforts in the world of practice” (Meyers, Durlak, et al., 2012, p. 464). Deriving from the QIF, Meyers and colleagues developed what they referred to as a”practical implementation tool” and the Quality Implementation Tool. The aim was to assist practitioners and those providing support to practitioners in implementing interventions with better quality (Meyers, Durlak et al., 2012; Meyers, Katz et al., 2012). However, efforts like these with the explicit goal of narrowing the gap between the science and practice of implementation may not be sufficiently practice‐friendly or ready to use. We do not know this because studies regarding their utility and usability do not exist.

In many ways, making use of implementation science knowledge could be viewed as an important implementation strategy with the potential to reduce the knowledge‐practice gap in health care. However, studies are needed to explore and assess this assumption. We strongly recommend research efforts focusing on further development of the concept of”practical implementation science.” There is a need for research on the applicability and use of models and frameworks as well as additional focus on the question of how to develop and evaluate more user‐friendly tools.

The rapidly growing body of evidence for implementation has the potential to bridge the knowledge‐practice gap in health care. However, implementation science knowledge is still predominantly in the domain of researchers. For knowledge on implementation to facilitate bridging the knowledge‐practice gap, it needs to be translated to user‐friendly tools that are actually used by healthcare practitioners.

With this editorial, we hope to have raised awareness of the need for the implementation science society to reflect upon the question of how we can support the systematic use of implementation science knowledge among leaders and other practitioners in healthcare settings.

Implementation science was born out of a desire to bridge the knowing‐doing gap (i.e., the gap between what is known and what is actually done in health care). It is a paradox if the knowledge produced in this field fails to reach the world of practice. For the practice of implementation to be furthered, we as researchers have an obligation to contribute to improved utilization and translation of the knowledge produced in the implementation science field.
